# Spatial and Temporal Evaluation of Ecological Footprint Intensity of Jiangsu Province at the County-Level Scale

**DOI:** 10.3390/ijerph17217833

**Published:** 2020-10-26

**Authors:** Decun Wu, Jinping Liu

**Affiliations:** 1 School of Public Administration and Sociology, Jiangsu Normal University, Xuzhou 221116, China; decunwu@jsnu.edu.cn; 2 School of Economics and Management, China University of Mining and Technology, Xuzhou 221116, China

**Keywords:** ecological footprint, ecological footprint intensity, Moran’s I, local spatial association index, micro-level, Jiangsu

## Abstract

Due to the high ecological pressure that exists in the process of rapid economic development in Jiangsu Province, it is necessary to evaluate its ecological footprint intensity (EFI). This article focuses on ecological footprint intensity analysis at the county scale. We used county-level data to evaluate the spatial distributions and temporal trends of the ecological footprint intensity in Jiangsu’s counties from 1995 to 2015. The temporal trends of counties are divided into five types: linear declining type, N-shape type, inverted-N type, U-shape type and inverted-U shape type. It was discovered that the proportions of the carbon footprint intensity were maintained or increased in most counties. Exploratory spatial data analysis shows that there was a certain regularity of the EFI spatial distributions, i.e., a gradient decrease from north to south, and there was a decline in the spatial heterogeneity of EFI in Jiangsu’s counties over time. The global Moran’s index (Moran’s I) and local spatial association index (LISA) are used to analyze both the global and local spatial correlation of EFIs among counties of Jiangsu Province. The high-high and low-low agglomeration effects were the most common, and there were assimilation impacts of counties with strong agglomeration on adjacent units over time. The results implied the utility of differentiated EFI reduction control measures and promotion of low-low agglomeration and suppression of high-high agglomeration in EFI-related ecology policy.

## 1. Introduction

The increasing greenhouse gas emissions and related climate change threats [1] are currently urgent global ecological issues, e.g., it was reported that there has been a 1.5 °C temperature increase compared to preindustry global levels [2]. With rapid urbanization processes and population growth occurring, land resources are also threatened [3]. China, as one of the fastest-growing countries undergoing economic development, urbanization and industrialization, is facing high carbon dioxide emissions and unsustainable land use changes, which are among the most serious ecological issues. China is responsible for global carbon emission reduction and its own needs for the construction of ecological civilization, and ecological sustainability should be taken into consideration in the process of economic development. Although a few studies have found that there are inverted-U relationships under the environmental Kuznets curve (EKC) model [4,5] under certain conditions, economic development has an obviously positive effect on the increase in carbon emissions [6] or ecological footprint [7] for most countries. Under the demand of maintaining economic development, reducing pollution intensity is a notable process to gradually move towards sustainable development, i.e., decreasing intensity of carbon usage has been listed as China’s future plan for the construction of an ecological civilization.

The pollution intensity refers to the ratio of pollution amount to the corresponding gross domestic product (GDP) in the process of production. Pollution intensity [8] is a worthwhile research point, and it is an important index to study for environmental policy making [9]. There is a trend in research to study environmental sustainability both qualitatively and quantitatively [10]. Meanwhile, environmental indicators play important roles in measuring environmental sustainability [11]. With increasing importance of greenhouse issues, carbon emissions are one of most important environmental indicators; therefore, carbon intensity is one of the most widely used indicators in the field of pollution intensity.

**Figure 1:**
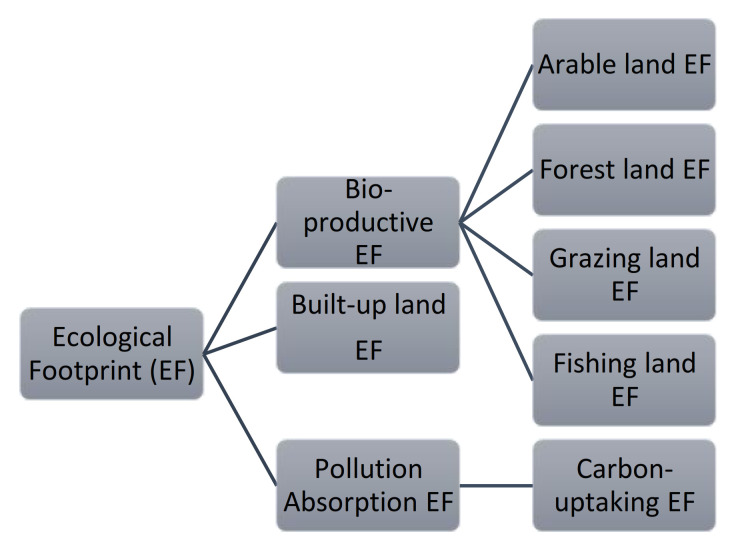
Classification structure chart of ecological footprint.

The ecological footprint (EF) is one of the most prominent ecological indicators and sustainability evaluation tools [12,13,14]. The EF is categorized by land type, and there are six types of ecological footprints: arable land EF, forestland EF, grazing land EF, fishing land EF, built-up land EF and carbon-uptaking land EF (Carbon EF hereinafter). The first four types of EFs are bio-productive EFs, as shown in Figure 1, all of which come from agricultural production. EF is an efficient environmental tool to evaluate environmental sustainability, e.g., it was used in analyzing the environmental consequences of household consumption for sustainable urban development [15]. It was also applied in the sustainable analysis of global marine fisheries and it is urgent to reduce the global fishing footprint for fishing sustainability [16]. Carbon EF is the only one pollution absorption EF. Compared to the carbon indicators, there are many advantages of the EF: (1) ecological footprint analysis (EFA) provides environmental thresholds in the assessments of environmental sustainability. EFA consists of two parts: EF and biocapacity (BC), accounting for the demand side and supply side of ecological resources, respectively. The EF and BC are grouped together as a set of ecological sustainability indicators, which could be used for judging the state of sustainability by comparing the values of EF and BC. (2) EFA integrates both carbon emissions and land ecology in a united land unit, which are two important environmental issues in the process of modern human development, and they are both critical factors influencing climate change [17]. As an environmental model, there are criticisms on the sustainable measurement function of EF [18], however, EF has been indeed playing an important role in the sustainable evaluation in practice [15,16] and has been becoming a policy-planning tool for making ecological environmental policies [19]. What is more, there are a few interesting research and conclusions combining the spatial factors in EF empirical studies and there is interesting policy implication. McDonald et al. [20] analyzed the EF of New Zealand’s land and found that there is an ecologically dependent relationship on EF. Zambrano-Monserrate et al. [21] used spatial Durbin to study the spatial correlation relationship among 158 countries, and there were significant spatial effects on EF. 

Initially, EFAs were mainly applied at the national scale [22], e.g., the Global Footprint Network (GFN) issues national footprint reports every year. After a series of amendments to ecological footprint accounting, EFAs were gradually applied at multiple geographic scales: the global scale, subnational scale, provincial scale and subprovincial scale [23]. The study of large-scale EF plays an important role in macroecological issues, especially in studies of greenhouse effects. However, when constructing operational environmental policy using EFA, large-scale EF research lacks corresponding local ecological details. Moreover, in consideration of the spatial relationship, large-scale EF research will conceal the spatial heterogeneity and spatial correlation of EF. With more detailed ecological data, small-scale EF research has more advantages than large-scale EF research on the aforementioned issues. 

It is a tricky issue to conduct EFA on a small-level geographic scale in standard EF accounting because it is complicated to collect corresponding statistical data in small-scale regions [24]. Nevertheless, there have been a few EF studies at a small scale after a few amendments to the accounting method [25,26,27]. Gottlieb et al. [28] conducted an EFA of a campus. Li et al. [29] also accounted for a campus’s EF with statistical consumption data and questionnaires. Hopton et al. [24] used small-level data and scaled upper-level data to construct a simple EF account. Ghosh et al. [30] constructed a cropland footprint from the perspective of accounting for corresponding consumption data. It is feasible to conduct microlevel EFA with consumption data instead of standard national footprint accounting (NFA) EF, which normally accounts for imported goods, exported goods and local production-related EF in a nation.

For the study of pollution intensifies, most studies are focused on carbon intensity [31], and the study of the intensity of ecological footprint (EFI) is relatively rare. As EFA integrates carbon emissions and land ecology as discussed above, using EFI has more advantages than carbon intensity in the study of sustainability issues. The EFIs were used in the study of EF inequality [32] and national EFI shifts [33]. To demonstrate the meaning of EFI in sustainability, it is worthwhile to conduct EFI analysis in small-scale regions. As Jiangsu Province is one of the most developed and typical provinces in China, the study presents a demonstration of Jiangsu to conduct EFA on a county-level scale, to illustrate the details of the accounting of EF and EFI and to analyze the spatial and temporal implied information of the EFIs in Jiangsu’s counties. Therefore, the following investigation was completed.

The article is organized as follows: Section 2 presents the study area and data source of this study. In Section 3, the accounting method of the ecological footprint and its intensity will be presented, and the methods of global spatial autocorrelation (Moran’s I) and local spatial autocorrelation (local spatial association index) will be illustrated, which will be used in the spatial analysis of EFIs of Jiangsu’s counties. In Section 4, temporal changes, spatial distribution and spatial autocorrelation of EFIs for Jiangsu’s counties are analyzed. Section 5 presents the policy implications for Jiangsu Province. Section 6 presents the conclusions based on the empirical analysis.

## 2. Study Area and Data Source

### 2.1. Study Area

**Figure 2:**
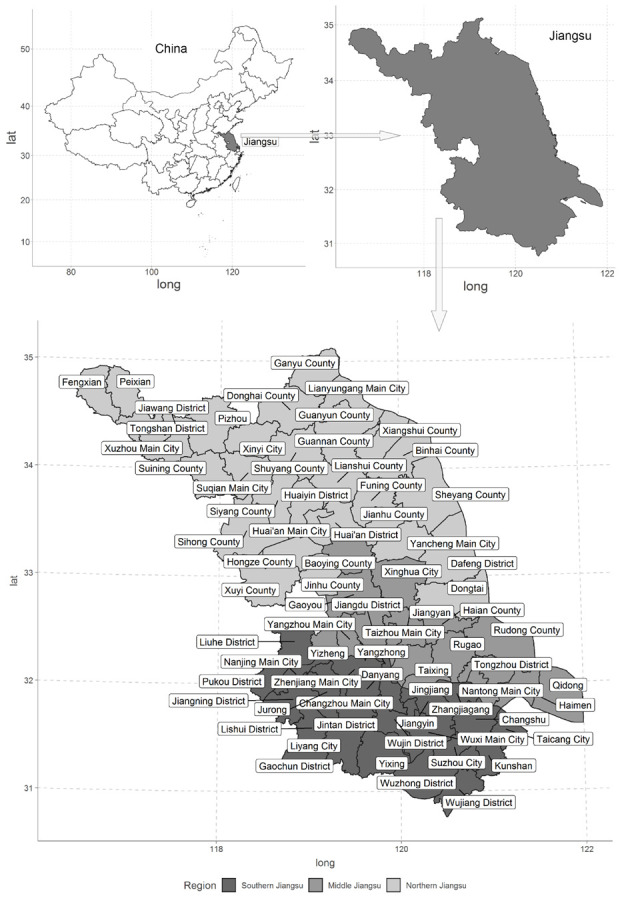
Administrative divisions of Jiangsu Province.

Jiangsu Province is located in eastern China. Based on the geographic location, Jiangsu Province is divided into three areas: southern Jiangsu, middle Jiangsu and northern Jiangsu, as shown in Figure 2. Jiangsu Province consists of 13 prefecture-level cities, i.e., Nanjing, Suzhou, Wuxi, Changzhou, Zhenjiang, Yangzhou, Taizhou, Nantong, Xuzhou, Lianyungang, Huaian, Suqian and Yancheng. In recent years, some of the original counties or county-level cities have merged into the urban area and have become a district; however, the economy and social development of this kind of district has not truly integrated into the main urban area, and we still consider them as a dependent country-level unit. Generally, a prefecture-level city consists of districts, counties and county-level cities. For example, Xuzhou consists of Xuzhou Main City, Tongshan District, Jiawang District, Fengxian County, Peixian County, Shuining County, Pizhou City and Xinyi City. Based on the data availability and the above reasons, Jiangsu is divided into 73 county-level administrative divisions in this study.

### 2.2. Data Source

The accounting of the ecological footprint involved the consumption of products from agriculture, forestry, animal husbandry, fishing and consumption of energy and built-up land use. As the analytical unit of this study is county-level, more data sources and transfers are needed to meet the data accuracy requirements. The main data sources are “China County Statistical Yearbook [34]”, “China City Statistical Yearbook [35]”, “Jiangsu Statistical Yearbook [36]”, statistical yearbooks of prefecture-level cities and some existing districts and counties [37,38] and annual statistical bulletins of national economic and social development [39]. The average output data of the global production of various agricultural products come from the FAO. Linear interpolations were used in some counties due to data unavailability. Remote sensing and geospatial data were used to account for the built-up EF.

## 3. Methods

### 3.1. Ecological Footprint Accounting

As EF is a consumption-side ecological indicator, national footprint accounting (NFA) [13,40] is normally applied on a large scale, e.g., the national scale. NFA is calculated by local production and import and export production, which is relatively convenient to account. However, this method is not suitable for county-level scale EF accounting. It is difficult to obtain the details of international and intercounty domestic import and export trade products on a county-level scale [41]. 

The following will integrate data from the past years of statistical data and remote sensing data to achieve the goal of research problems and use various types of data in Jiangsu Province to analyze the biological production EF, built-up land EF and carbon EF accounting model applicable to Jiangsu Province and district/county scales.

The bio-productive EF is calculated by the following equation:

(1) $${\mathrm{EF}}_{\mathrm{bio}}=\underset{i}{\sum }\frac{C_{i}}{Y_{i}}\cdot {\mathrm{YF}}_{i}\cdot {\mathrm{EQF}}_{i}$$

where $C_{i}$ is the annual total consumption of product i, and ${\mathrm{YF}}_{i}$ indicates the yield factor of the specific land type of product i, which represents the ratio of local average yield to world yield. To make EF accounting more accurate, the yield factor for each year was calculated instead of using a fixed value. ${\mathrm{EQF}}_{i}$ represents the equivalence factor, which could transfer different types of land to world average land measured by land productively.

The built-up EF is calculated by the following equation:

(2) $${\mathrm{EF}}_{\mathrm{built-up}}{=A}_{\mathrm{built}\_\mathrm{up}}\cdot {\mathrm{YF}}_{\mathrm{arable}}\cdot {\mathrm{EQF}}_{\mathrm{arable}}$$

where $A_{\mathrm{built}\_\mathrm{up}}$ is the built-up land area for a county measured by hectares, and ${\mathrm{YF}}_{\mathrm{arable}}$ and ${\mathrm{EQF}}_{\mathrm{arable}}$ refer to the yield factor and the equivalence factor of arable land, respectively. Since built-up land is generally arable, the two factors are adopted in the built-up land EF.

The carbon EF is calculated by the following equation:

(3) $${\mathrm{EF}}_{\mathrm{carbon}}=\frac{E_{c}\left(1-S_{ocean}\right)}{Y_{c}}\cdot {\mathrm{EQF}}_{\mathrm{forest}}$$

where $E_{c}$ refers to the carbon dioxide and equivalent emissions consumed in a county, $S_{ocean}$ refers to the proportion of the ocean that attracts global human carbon emissions, and the proportion was taken as 28% in this study [40], $Y_{c}$ refers to the global average carbon absorption capacity of forests, and ${\mathrm{EQF}}_{\mathrm{forest}}$ is the equivalence factor of forestland. Since the main carbon absorption sources are forestland, the EF model adopts forest EQF in calculating carbon footprints.

### 3.2. Ecological Footprint Intensity

Ecological footprint intensity is a similar concept to the concept of carbon intensity and is measured by the ratio of gross EF to GDP, as defined in Equation (4). Similarly, the EFI can be analyzed by each land type of EF. The concepts of arable land EFI, forestland EFI, grazing land EFI, fishing land EFI, built-up land EFI and carbon-uptaking land EFI can be used in the following. Each type of EFI could be accounted for within the gross EFI by the ratio of the quantity of a type of EF to GDP.

(4) $$\mathrm{EFI}=\frac{EF}{GDP}=\frac{EF_{carbon}+EF_{bio}+EF_{built-up}}{GDP}={\mathrm{EFI}}_{\mathrm{carbon}}+{\mathrm{EFI}}_{\mathrm{bio}}+{\mathrm{EF}}_{\mathrm{built-up}}$$

### 3.3. Spatial Autocorrelation Analysis

#### 3.3.1. Global Spatial Correlation Index

Moran’s index (Moran’s I) is a global spatial autocorrelation index. As a single index, Moran’s I calculates the degree of spatial association of each unit in the study area by adding spatial relationship information. Moran’s I is used to measure the overall spatial correlation for a studied area, and it is widely applied in multiple disciplines for its concision. The global Moran’s I is calculated as follows [42]:

(5) $$I=\frac{\sum_{i=1}^{n}\sum_{j\neq i}^{n}w_{ij}\left(X_{i}-\bar{X}\right)\left(X_{j}-\bar{X}\right)}{S^{2}\sum_{i=1}^{n}\sum_{j\neq i}^{n}w_{ij}}$$

where $S^{2}=\frac{1}{N}\sum_{i}{\left(X_{i}-\bar{X}\right)}^{2}$, $\bar{X}=\frac{1}{N}\overset{n}{\underset{i=1}{\sum }}X_{i}$, N is the sample number, and $w_{ij}$ represents the adjacent relationship between region i and j, which is defined as follows:

(6) $$w_{ij}=\left\{\begin{array}{cc} 1 & if\;sample\;i\;and\;sample\;j\;are\;adjacent\\ 0 & other\;scenario \end{array}\right.$$

The spatial matrix of adjacent relationships is constructed when all adjacent relationships are judged. There are a few algorithms to judge the adjacent relationship, including k-nearest neighbors, rook contiguity and queen contiguity [42]. K-nearest neighbors consider the k-nearest neighbors as the existence of adjacent relationships. Rook contiguity is judged on edge adjacency, while queen contiguity added corner adjacency based on rook contiguity [43]. This study uses queen contiguity to construct a spatial weight matrix of adjacent relationships; meanwhile, the matrix will be normalized before further usage. The value of Moran’s index ranges from −1 to 1. The more the value tends to 1, the stronger the positive spatial correlation is. Otherwise, the less the value tends to −1, the stronger the negative spatial correlation is. 

The significance of the value of Moran’s index could be assessed by permutations in GeoDa [44]. The null hypothesis is that there is no spatial correlation. The permutations process randomly, permuting the observed values over the locations and then calculating a reference distribution [44]. The pseudo *p*-value is calculated based on the reference distribution to assess the significance, as shown below [44]:

(7) $$p=\frac{R+1}{M+1}$$

where R is the number of times for computation of the permuted data sets, and M is the number of permutations. To get a more precisely significant result for the global Moran’s I, 999 permutations should be conducted and a *p*-value of 0.001 should be considered as a significant result.

#### 3.3.2. Local Spatial Association Index

Based on the principle of global Moran’s index, Anselin [45] proposed the local spatial association index (LISA). Instead of the global autocorrelation degree of the spatial unit, the local spatial correlation characteristics are analyzed and presented by LISA. Anselin used local Moran’s I and Moran’s I scatter plots to perform spatial clustering analysis and outlier analysis. Local Moran’s I is decomposed from global Moran’s I. A local Moran’s statistic for a spatial unit i is expressed as [45,46]

(8) $$I_{i}=\frac{z_{i}}{s^{2}}\underset{j}{\sum }w_{ij}z_{j}$$

where $z_{i}$ and $z_{j}$ are the deviations from the mean, j indicates all the spatial adjacent units defined by the criteria of contiguity, and $s^{2}$ and $w_{ij}$ are defined the same as in Equation (5). The larger the positive value of local Moran’s I of a spatial unit, the more similar to the adjacent units it is. On the contrary, a negative value of local Moran’s I indicates heterogeneity to adjacent units.

In a Moran scatter plot, the points with the positive sign of $z_{i}$ lie on the right X-axis, and the points with the negative sign of $z_{i}$ lie on the left X-axis. Similarly, the points’ positions of the Y-axis are dependent on the signs of the spatial lag value for the points. The four quadrants of an axis would represent the H-H, L-H, H-L and L-H from the upper-right quadrant to the lower-right quadrant counterclockwise [45].

The significance test of local Moran’s I would be conducted by GeoDa’s Randomization of 999 permutations as the significance of the global Moran’s I, and a p-value less than the cut-off 0.05 could be regarded as a significant result [46]. Based on the result of local Moran’s I and the aggregation type classified by the Moran scatter plot, the LISA map could be drawn and filled with different colors to highlight each aggregation type.

## 4. Results

### 4.1. Temporal Changes in EFI for Jiangsu’s Counties

**Figure 3:**
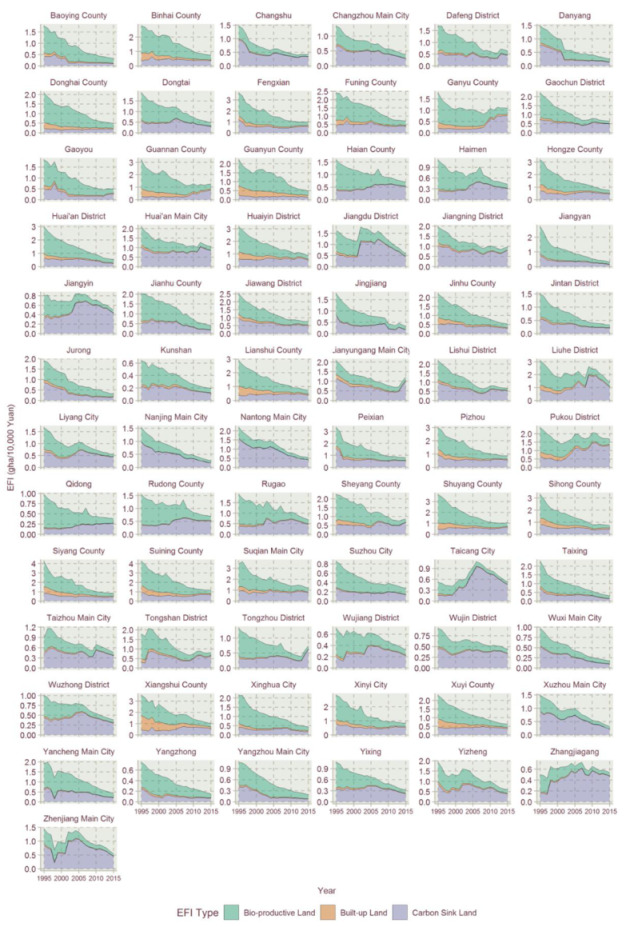
Temporal changes in EFIs of Jiangsu Province for 1995–2015, categorized by bio-productive land, built-up land and carbon sink land.

According to the EF and EFI accounting method, the EFs and corresponding EFIs of Jiangsu’s 73 county-level units were conducted from 1995 to 2015. The year 2000’s constant price was used to account for the EF and corresponding EFI of each county. The temporal changing trends of EFI of Jiangsu’s counties are shown in Figure 3. It is found that the EFIs of most counties showed an obvious downward trend over the past 20 years, and there were only two counties with a net increase in EFI, i.e., Zhangjiagang City and Taicang City in 2015, reaching 1.06 and 1.02 times that of the values in 1995, respectively. In addition, Pukou District, Jiangyin City and Ganyu District experienced the smallest decline, reaching 71.8%, 61.8%, and 61.6% of the values in 1995, respectively. In contrast, the EFI of Jiangyan District, Taixing City, Nanjing Main City, Jurong and Danyang declined the most in 2015, reaching 11.8%, 13.5%, 16.7%, 16.7% and 17.1% of the values in 1995, respectively. Temporal change analysis is demonstrated by two parts in this section, i.e., EFI proportional change analysis and EFI trend change analysis.

The EFI’s proportional structure varies among those counties. One part of the counties is dominated by the carbon EFI throughout the duration of the study, e.g., Changshu, Jiangyin and Jurong, Taichang and Zhangjiagang, most of which are economically developed counties. Other countries are dominated by bio-productive EFI during the timeperiod, e.g., Baoying County, Binhai County and Jianhu. Due to the change in economic structure, the EFI proportional structures of many counties changed during the years studied, and the main proportion changed from bio-productive EFI to carbon EFI. Sheyang County, Hongze County, Jintan District and Shuyang County were typical examples.

**Figure 4:**
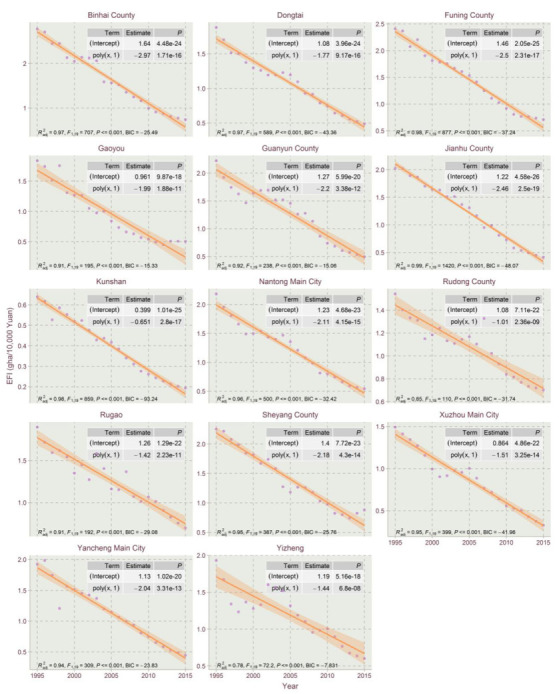
Linear orthogonal curve fitting of EFIs of Jiangsu Province for 1995–2015.

**Figure 5:**
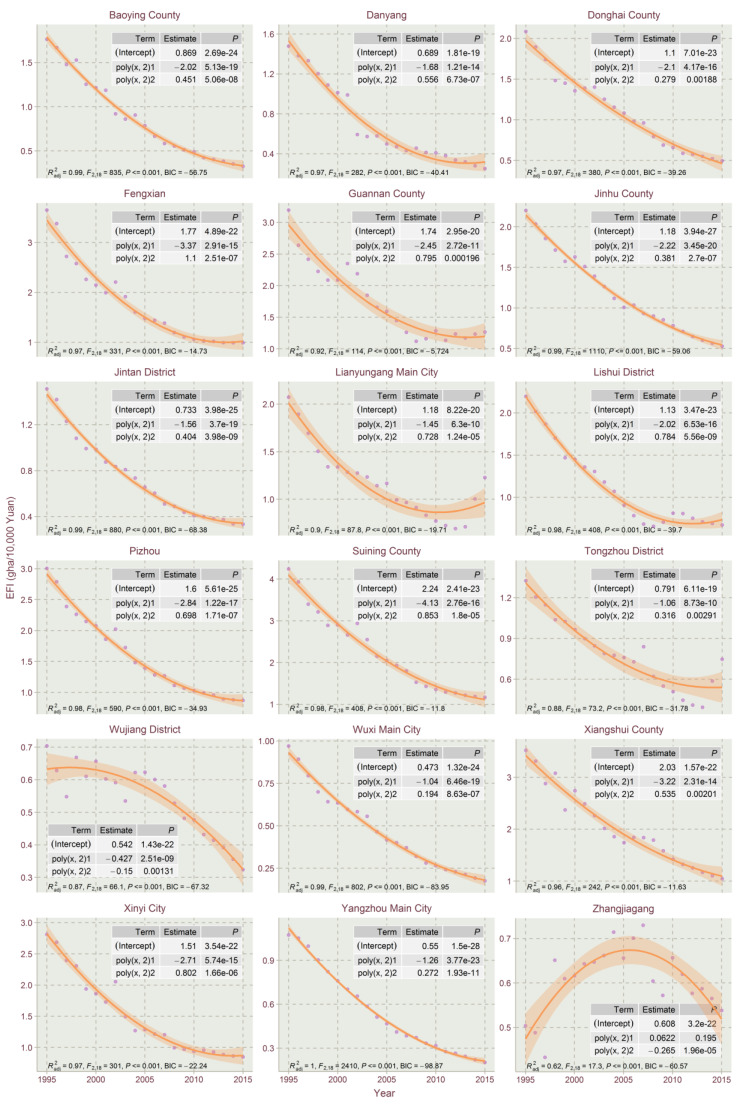
Quadratic orthogonal curve fitting of EFIs of Jiangsu Province for 1995–2015 (Part 1).

**Figure 6:**
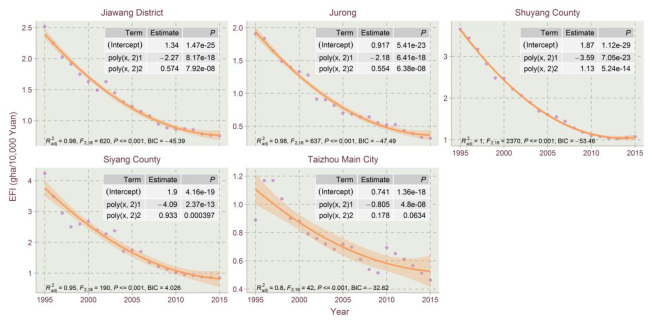
Quadratic orthogonal curve fitting of EFIs of Jiangsu Province for 1995–2015 (Part 2).

**Figure 7:**
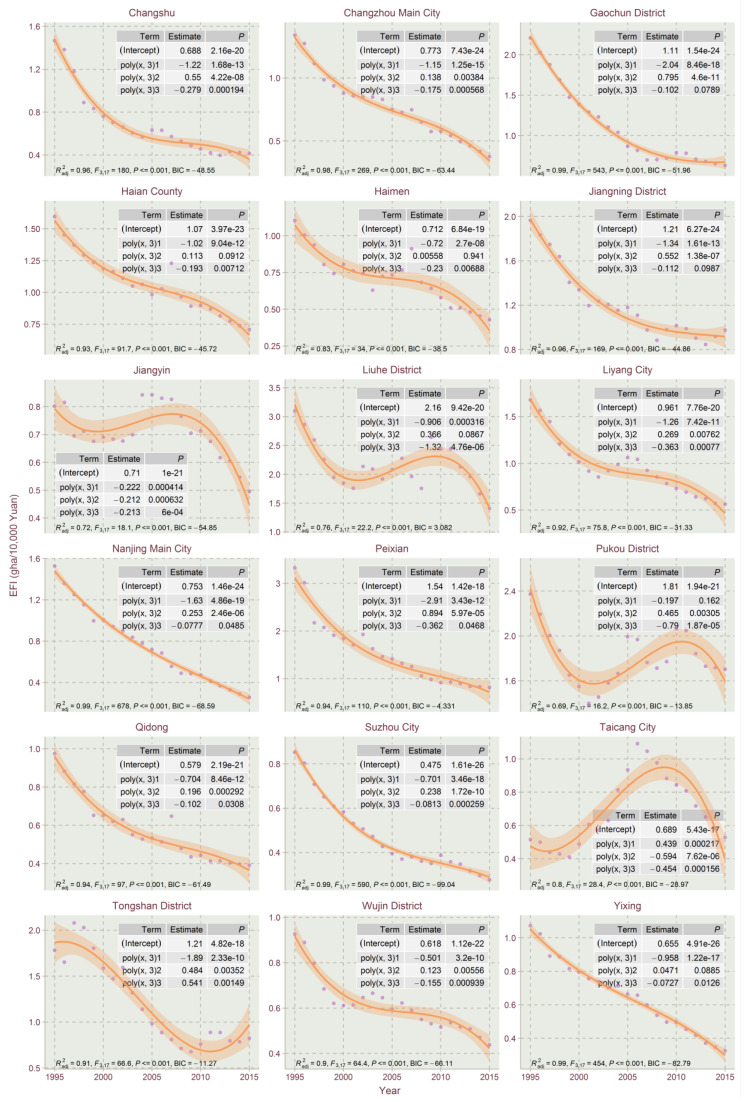
Cubic orthogonal curve fitting of EFIs of Jiangsu Province for 1995–2015 (Part 1).

**Figure 8:**
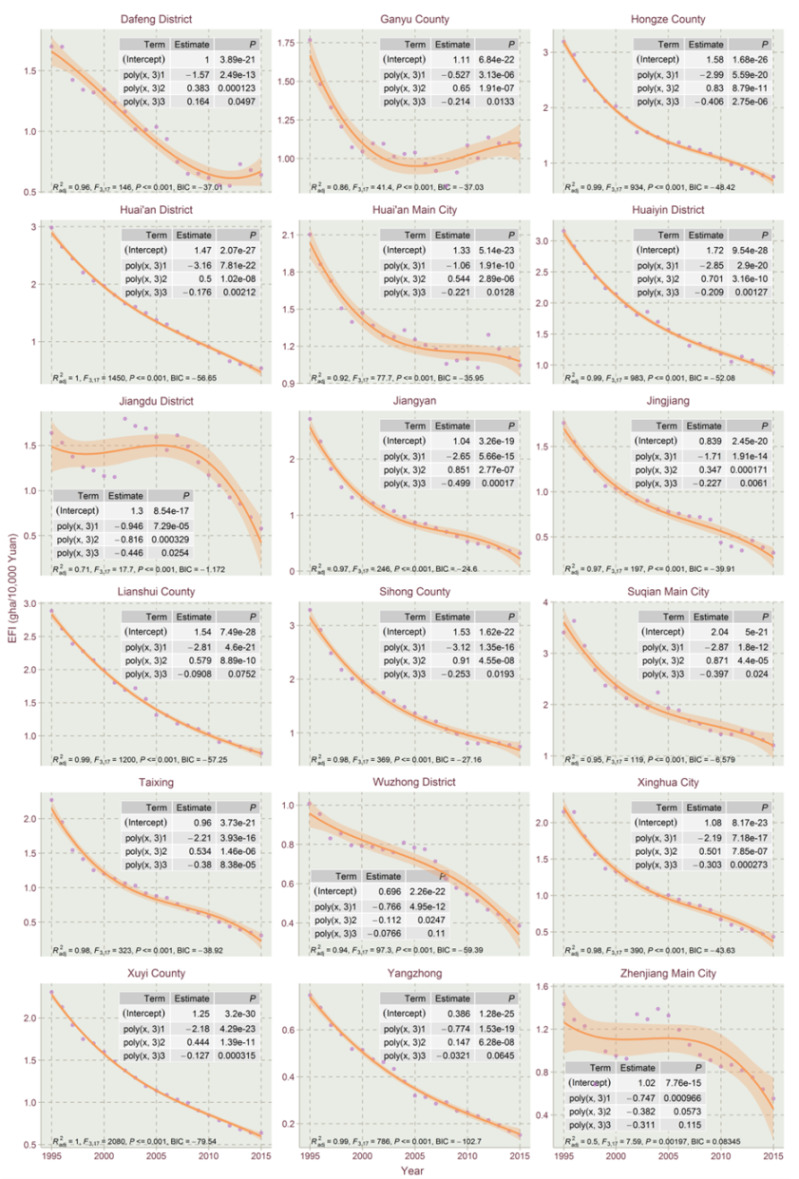
Cubic orthogonal curve fitting of EFIs of Jiangsu Province for 1995–2015 (Part 2).

The results show that the EFIs of most counties exhibited a declining trend in the studied time period, which could be explained by the high economic development and eco-friendly technology development in Jiangsu for the period. This paper used the orthogonal curve fitting method in R tools to capture the EFI temporal trends for Jiangsu’s counties. The linear model, the quadratic model and the cubic model of quadratic orthogonal curve fitting were conducted with R to select the best fitting model for the EFI temporal trends. The model selection criteria include the Bayesian information criterion (BIC), F-statistics, adjusted goodness of fit ($R_{adj}^{2}$) and the significance of the regression coefficient. From the curve fitting of EFIs’ timing changes, the EFI’s trend evolution of Jiangsu’s counties could be divided into five types: linear declining type, N-shape type, inverted-N type, U-shape type and inverted-U shape type. Due to the size of figures, the three types of fitting results are divided into Figure 4, Figure 5, Figure 6, Figure 7 and Figure 8.

As shown in Figure 4, a few counties (14 total) in Jiangsu belong to the linear declining type, which presented declining EFI trends during the entire study period. This type of county includes Binhai County, Dongtai, Funing County, Gaoyou, Guanyun County, Jianhu County, Kunshan, Nantong Main City, Rudong County, Rugao, Sheyang County, Xuzhou Main City, Yancheng Main City and Yizheng, most of which are located in northern Jiangsu. 

The EFI trends of the U-shaped type first declined and then showed a slightly increasing trend. As shown in Figure 5 and Figure 6, with the positive coefficient of quadratic orthogonal regression, there are 21 counties in Jiangsu for this type, i.e., Baoying County, Danyang, Donghai County, Fengxian, Guannan County, Jinhu County, Jintan District, Lianyungang Main City, Lishui District, Pizhou, Suining County, Tongzhou District, Wuxi Main City, Xiangshui County, Xinyi City, Yangzhou Main City, Jiawang District, Jurong, Shuyang County, Siyang County and Taizhou Main City. Among them, the EFI trend of Lianyungang Main City has the most obvious U-shape, which should be given more attention.

The inverted-U shape type featured EFI inclining first and declining thereafter. As shown in Figure 5, there are only two counties in Jiangsu of this type, Wujiang District and Zhangjiagang, which are typical counties that changed their economic development model at the turning point which resulted in EFI declining rapidly.

The N-shape type is characterized by EFI rising first, then dropping, and finally climbing, with two counties in Jiangsu belonging to the type, i.e., Tongshan District and Dafeng District.

The inverted-N type is characterized by EFI dropping first, then rising, and finally falling, with 34 counties in Jiangsu belonging to the type, i.e., Changshu, Changzhou Main City, Gaochun District, Haian County, Haimen, Jiangning District, Jiangyin, Liuhe District, Liyang City, Nanjing Main City, Peixian, Pukou District, Qidong, Suzhou City, Taicang Cit, Wujin District, Yixing, Ganyu County, Hongze County, Huai’an District, Huai’an Main City, Huaiyin District, Jiangdu District, Jiangyan, Jingjiang, Lianshui County, Sihong County, Suqian Main City, Taixing, Wuzhong District, Xinghua City, Xuyi County, Yangzhong and Zhenjiang Main City. A lot of them are economically developed counties. Jiangdu district, Liuhe District and Pukou District present the most obvious inverted-N fitting trends due to their economic structure and zoning adjustment in the studied time period. Most of them exhibited slightly inverted-N curve characteristics, tending to steady EFI dropping.

The temporal trends of the linear declining type and most of the inverted-N types could be regarded as declining type as those inverted “N” trends are not obvious. 

The reasons why the temporal trends in EFI for those 73 counties varied could be explained through their economic development levels and the EFI’s proportional structures of the whole cycle. 

Overall, the proportion of bio-productive EFI and built-up EFI shows declining trends for each county in Jiangsu from 1995 to 2015, however, the proportions of carbon EFI show varying trends for the 73 counties. For the bio-productive EFI and built-up EFI, in rapid economic development and industrialization of Jiangsu Province in 1995–2015, the bio-productive EFI and built-up EFI impact less and less on the total EFI. The form of varying temporal trends in EFI besides the declining type is mainly contributed by the varying trends of carbon EFI. The carbon intensity trends are mostly dependent on the economic development and industrial structure, and green technology, which has a significant impact on carbon intensity [47]. The counties of the linear declining type and most of the inverted-N type are of steady carbon EFI evolution, and normally showed a steadily declining trend, i.e., Binhai County, Dongtai, Funing County and Huan’an District.

Most of the U-shaped EFI temporal trends are featured with a high proportion of initial bio-productive EFI. Though the biological EFIs of those counties are declining fast, their proportion is getting smaller and smaller. Due to the notion that there is not too much room to drop for bio-productive EFIs, there is EFI climbing potential with the carbon EFI increase in the rapid economic development for counties such as Baoying County, Danyang, Donghai County, Fengxian and Jinhu County.

### 4.2. Spatial Distribution of EFI for Jiangsu’s Counties

**Figure 9:**
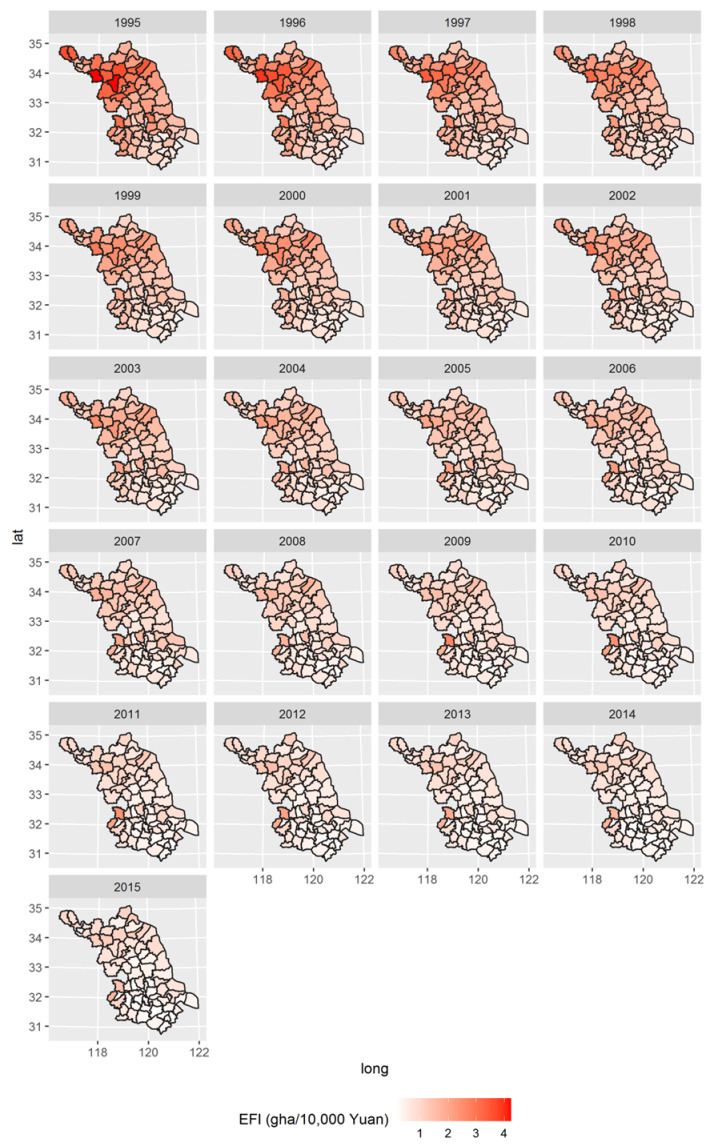
Spatial distribution of EFI of Jiangsu Province in 1995–2015.

The spatial distributions of Jiangsu’s county-level EFI from 1995 to 2015 are presented in Figure 9. On the whole, there is obvious spatial heterogeneity in the distribution of EFI. The feature of EFI’s distribution shifted greatly during the studied years. In 1995, the high EFI values were mainly concentrated in the northern Jiangsu area, where Suining, Sihong and Fengxian were the top three largest EFIs in Jiangsu, with values of 4.64, 4.24 and 3.65 gha/10,000 CNY, respectively. There was an obvious difference among the northern and southern areas, and the EFI distribution showed a gradient descent from north to south in 1995. As shown by the change in map color depth over time, the difference in the north–south gradient descent decreased. Over time, the spatial distribution of the EFI color depth appears increasingly evenly, indicating that the spatial heterogeneity of EFI has become small in recent years.

Due to the aggregation effect of the economy and eco-friendly technology, the EFIs of the main cities of the 13 prefecture-level cities in Jiangsu Province in each year are generally much smaller than those of the counties under their jurisdiction from 1995 to 2015, e.g., the EFI of Nanjing Main City is much lower than that of counties in its jurisdiction, including Pukou District, Jiangning District, Liuhe District, Lishui District and Gaochun District, in each year. Taking Nanjing City in 2015 as an example, the EFI of its main city is 0.255 gha/10,000 CNY, and the EFIs of its other administrative counties, Pukou District, Jiangning District, Liuhe District, Lishui District and Gaochun District, were several times higher than those of the former, reaching 1.704, 0.976, 1.412, 0.671 and 0.634 gha/10,000 CNY, respectively.

Overall, the spatial distributions of EFIs in Jiangsu assumed a certain regularity in the north–south direction in the timeline. More spatial features were researched with the spatial autocorrelation analysis shown below.

### 4.3. Spatial Autocorrelation Analysis of EFI for Jiangsu’s Counties

#### 4.3.1. Global Spatial Correlation of EFI

**Figure 10:**
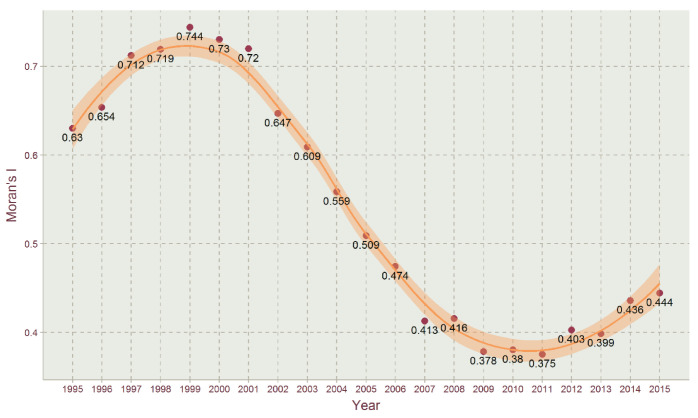
Temporal changes in Moran’s index (I) of Jiangsu’s EFI in 1995–2015.

With the adjacent method of queen contiguity, Moran’s I of Jiangsu’s EFI in 1995–2015 is shown in Figure 10. With the 999 permutations in GeoDA, all the pseudo *p*-values are 0.001, indicating these global spatial agglomeration effects are statistically significant. All the values of Moran’s I in the studied time period were greater than 0.35, indicating that there were significant positive spatial correlations among the EFIs of all districts and counties in Jiangsu during the studied period, and the spatial distribution of Jiangsu’s EFI shows a certain spatial agglomeration effect. Moreover, the correlation and agglomeration varied greatly among different years. The figure shows that the time-series curving line of EFI assumes an N-shape, and specifically, the value of Moran’s I peaked at 0.744 in 1999, which was an increase from 0.63 in 1995, and then it almost consistently declined with the lowest value of 0.375 in 2011; it slightly climbed to 0.444 in 2015. The results of Moran’s I demonstrate that the years 2005 and 2006 are the dividing line, the higher spatial correlations and spatial agglomerations of EFI were concentrated in 1995–2005, with Moran’s I more than 0.5, and the lower ones were concentrated in 2006–2015, with Moran’s I less than 0.5. With the increased disparity of economic development among southern Jiangsu, middle Jiangsu and northern Jiangsu, the spatial correlation and spatial agglomeration effect of the corresponding EFI dropped in recent years compared to the former years.

#### 4.3.2. Local Spatial Correlation of EFI

**Figure 11:**
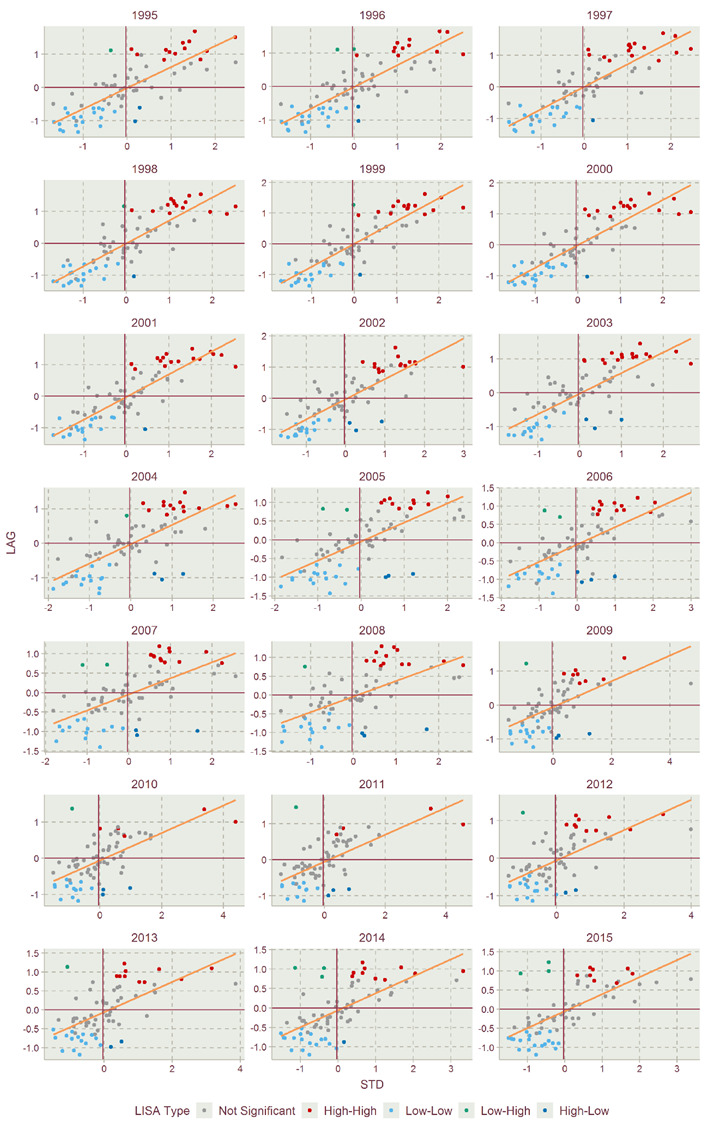
Moran’s scatter plots of EFIs of Jiangsu Province in 1995–2015.

With the statistical processing in 3.3.2, the Moran’s scatter plots of EFIs of Jiangsu Province in 1995–2015 were drawn as shown in Figure 11. In the Moran’s scatter plot for each year, the X-axis represents the standard deviation (STD), and the Y-axis represents the lag term (LAG). The coordinate axis is divided into four quadrants, i.e., quadrants I, II, III and IV, representing high-high (H-H) agglomeration, low-high (L-H) agglomeration, low-low (L-L) agglomeration and high-low (H-L) agglomeration, respectively. The H-H, L-H, L-L and H-L agglomerations denote the regions’ agglomerations with high, low, low and high EFI, adjacent to regions with high, high, low and low EFI, respectively.

**Figure 12:**
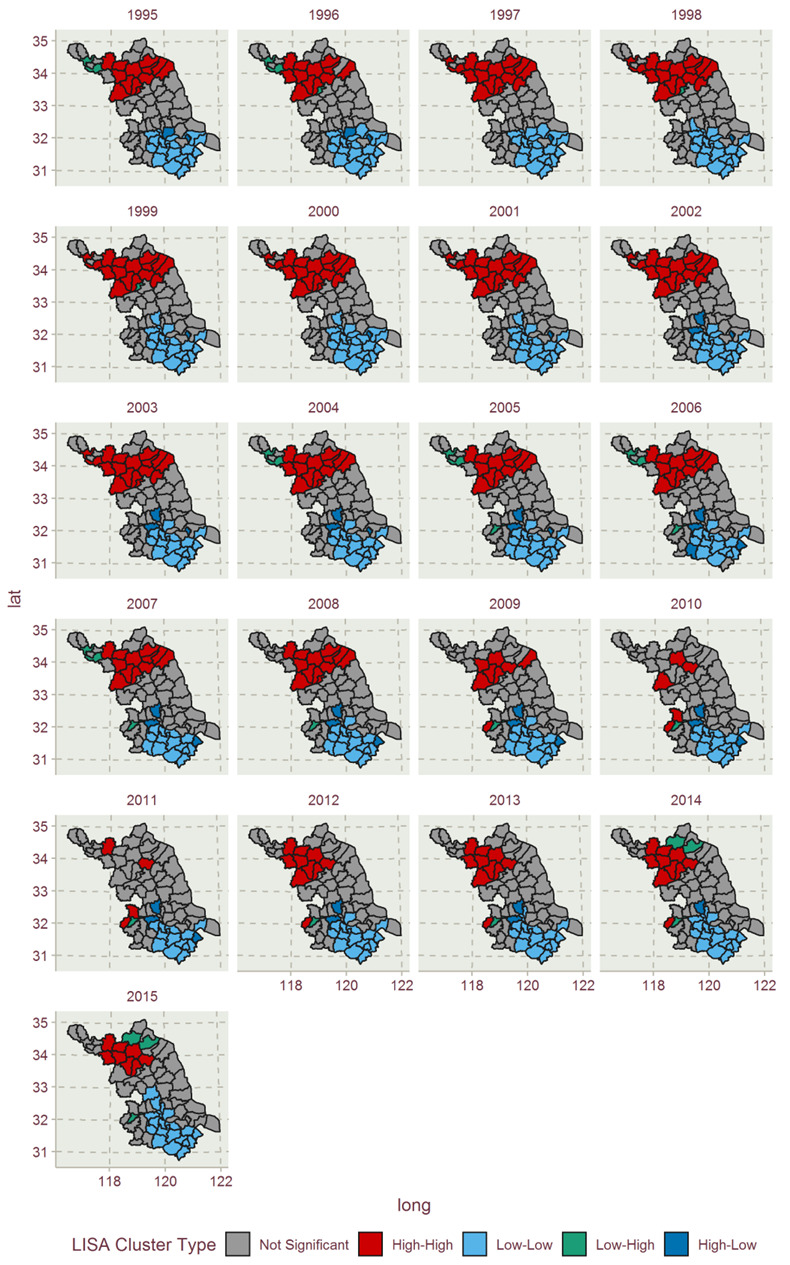
Spatial distribution of EFIs of Jiangsu Province in 1995–2015.

The gray points in the figure indicate that the local spatial autocorrelation of the districts and counties have not passed the 5% significance level test. These nonsignificant districts and counties are mainly concentrated in the H-H and L-L agglomeration types. The specific agglomeration types of EFI also can be presented in the LISA map, as shown in Figure 12, which were filled with the colors the same as the agglomeration types’ in Figure 11.

Figure 12 shows the LISA cluster maps of Jiangsu in 1995–2015. There are one-to-one correspondences with the subfigures between Figure 11 and Figure 12. Figure 12 shows the spatial agglomeration maps of the EFI of various districts and counties in Jiangsu Province from 1995 to 2015. The space units marked with colors in the figure indicate that they have passed the 5% significance agglomeration test. The study found that in all years, H-H agglomeration and L-L agglomeration types account for the majority, and H-L agglomeration and L-H agglomeration spatial units are few and relatively scattered. From 1995 to 2011, H-H agglomeration showed a downward trend, and from 2012 to 2015, the H-H agglomeration trend increased from the 2011 value. The number of L-L agglomerations changed little during the study year.

H-H agglomeration type: this type is mainly located in northern Jiangsu during the study period. In 1995, there were 13 units: Binhai County, Guannan County, Guanyun County, Huai’an Main City, Huaiyin District, Lianshui County, Pizhou, Shuyang County, Sihong County, Siyang County, Xiangshui County, Xinyi City and Suqian Main City. Over time, the number of counties in the district reached a maximum of 16, occurring in 1997, 2000, 2001 and 2003, and the number gradually declined until 2012. The specific changes in 2001 include Suning, Tongshan and Funing as the agglomeration types, these districts and counties and Huai’an Main City. By 2005, it gradually became insignificant and no longer fell into this category. By 2011, greater changes had taken place, and all original types except Lianshui and Pizhou disappeared from the type; however, some districts entered this type, such as Liuhe District and Pukou District. This type of agglomeration gradually began to appear in southern Jiangsu. In 2015, this type reached a relatively high number of agglomerations in the northern Jiangsu area, which increased to nine.

L-H agglomeration type: this type of agglomeration district and county is relatively small and scattered. From 1995 to 2013, the number of districts and counties was between zero and two and reached a maximum of three in 2014–2015. The districts and counties that appeared in these years are Donghai County, Guanyun County, Huai’an Main City, Tongshan District and Nanjing Main City, and these districts and counties show the distribution characteristics of low EFI and neighboring high EFI. Nanjing showed this type of agglomeration from 2005 to 2015, and other districts and counties have seen fewer years. 

L-L agglomeration type: the number of agglomeration counties of this type has the largest proportion of all significant agglomeration types, and the geographic units in each year are mainly concentrated in southern Jiangsu. In 1995, 18 districts and counties belonged to this type, namely Changshu, Changzhou Main City, Danyang, Haimen, Jiangyin, Jintan District, Jingjiang, Kunshan, Suzhou City, Taicang City, Tongzhou District, Wuxi Main City, Wujiang District, Wujin District, Yangzhong, Yixing, Zhenjiang Main City and Wuzhong District, three counties of which belong to middle Jiangsu, and the others of which are from southern Jiangsu. In 2000, the number of this agglomeration type peaked for the studied time period at 21, and Jiangdu, Zhangjiagang and Taixing entered this type in 2000. Since 2001, the number of districts and counties of this type of agglomeration has gradually declined in fluctuations, and in 2011, the number fell to 12. The number has gradually risen and rebounded to 19 in 2015. From 1995–2015, this type of district and county had different characteristics from 1995 to 2015. Changshu, Changzhou Main City, Danyang, Jiangyin, Kunshan, Suzhou City, Wujin District, Wuxi Main City, Wuzhong District and Yixing have always belonged to the L-L agglomeration type during the studied period, which could be regarded as the core agglomeration members of this type. Wujiang District and Jintan District were absent from the agglomeration for only one–two years, 2006 for the former, and 1997 and 2011 for the latter. Haimen did not belong to the L-L type from 2006 to 2011, when it belonged to the H-L agglomeration type, indicating that its EFI is significantly higher than its adjacent area. Yangzhou was not within the L-L agglomeration member list from 2002 to 2012, and Taixing and Zhenjiang Main City had multiple changes in the existence of the L-L agglomeration state. Jingjiang, Liyang City, Jiangdu District, Tongzhou District, Taizhou Main City, Rugao, Gaoyou, Yangzhou Main City and Zhangjiagang were part of the L-L agglomeration group for only a few years, indicating that they are not stable in maintaining a low EFI state.

H-L agglomeration type: this type of agglomeration area has a small number of counties and is relatively scattered, mainly in the middle Jiangsu area. The change trend of the number is in an inverted-U trend. From 2002 to 2011, the number was between three and four and only one and two before 2001 and after 2012, respectively. The number of this type dropped to zero in 2015. Jiangdu District, Zhenjiang Main City and Nantong Main City belong to this type for more than 10 years and are the core members of this type. Taicang City, Taixing and Liyang City only belonged to this type for a few years. 

The agglomeration type of a county tends to change more easily when the quantities of geographic units are too low for the agglomeration type, e.g., the agglomeration types of Tongshan and Nanjing Main City changed many times during 1995–2015, as they were alone. There were also assimilation impacts of counties with strong agglomeration on adjacent units.

From the perspective of spatial geography, the spatial agglomeration effect of the economy, society and resources is caused by the spatial correlation, where everything has a certain spatial correlation, thus the closer the geographical distance between things, the stronger the spatial correlation [48]. The formation of agglomeration is a process that economic entities seek to collaborate between and reduces the cost of production, and it is mostly an economic process. Each EFI agglomeration type in this study was driven by the economic development, ecological resource structure and industrial structure and green technology. That the counties were mostly gathered in northern Jiangsu could be explained by its relatively lower level of economic development, the relatively lower proportion of tertiary industry and agglomeration of highly polluting enterprises. For example, due to the relatively rapid economic development and the introduction of high-tech and low-carbon industries in northern Jiangsu in 2005–2011, the H-H agglomeration effect was getting weaker and weaker. As southern Jiangsu and parts of middle Jiangsu have a more developed economy and more agglomerations of high-tech and green technology industries, the L-L EFI agglomeration types were mainly gathered there.

## 5. Policy Implications

The ecological aim was to restrain the EFI for each county in Jiangsu. Based on the results analysis of spatial and temporal evaluation and the spatial autocorrelation analysis of Jiangsu’s EFI, the following policy implications are presented:

(1) As there were obvious heterogeneities of the EFI’s evolution in Jiangsu’s counties, the differentiated EFI reduction control measures should be carried out. For example, the counties with U-shape and N-shape EFI trends, and the high EFI (low EFI declining rate) should be strength regulations. The typical counties for the former are Dafeng District, Guannan County, Lianyungang Main City, Tongshan District and Tongzhou District. The typical counties for the latter are Huai’an Main City, Haian County, Sheyang County, Rugao City and Suqian City, all of which have EFIs with a high value and low declining rate.

(2) The H-H EFI agglomeration regions are those with the main EFI restraining regulations, and it is necessary to suppress the EFI of those counties and reduce their external influence on neighbors. The ecologically friendly advantage of L-L EFI agglomeration regions should be strengthened and spread out, helping H-L and L-H turn to L-L EFI agglomeration. Specifically, these strategies should be enacted on the core members, e.g., Changshu, Changzhou Main City, Danyang, Jiangyin, Kunshan, Suzhou City, Wujin District, Wuxi Main City, Wuzhong District and Yixing, for L-L agglomeration.

## 6. Conclusions

This paper studied the accounting of the ecological footprint and ecological footprint intensity at the county-level scale. As mapping is of great significance in ecological research [49], we used Moran’s I and GIS mapping to reveal the temporal and spatial changes in the EFI of Jiangsu Province at the county-level scale. The EFI of Jiangsu’s 73 counties during 1995–2015 was accounted for, and the trends, spatial distribution and spatial autocorrelation of those EFIs were analyzed. The reasons for the existence of differences in temporal trends and agglomeration types were analyzed. This paper aimed to explore the trends of spatial and temporal evaluation of EFI in Jiangsu’s counties in order to be a reference for low EFI policy making for each specific county. The specific conclusions are as follows:

(1) There are obvious differences in the temporal change characteristics of the ecological footprint intensity among counties in Jiangsu Province. Its inner structure feature could be well explored through a smaller scale with the GIS color maps, and it was found that there was a certain regularity of EFI spatial distributions, i.e., gradient descent from north to south, and there was a declining spatial heterogeneity of EFI in Jiangsu’s counties over time. 

(2) Through linear, quadratic and cubic orthogonal curve fittings, the development trend of the EFI of each county in Jiangsu Province could be well typed and summarized. Then, different EFI restraining administrations for different types of counties could be carried out. The empirical analysis concludes that there are five types of EFI evolution trends in Jiangsu: linear declining type, N-shape type, inverted-N type, U-shape type and inverted-U shape type. The linear declining type and most inverted-N types could be seen as the steadily declining types. Although most parts of Jiangsu’s counties exhibit declining EFI trends, the EFIs of a few counties present increasing trends or barely declining trends. The reason for the formation of different EFI temporals is contributed by the economic development levels and the EFI’s proportional structures of a county. 

(3) In terms of studying spatial correlation, when using the Moran’s I and local Moran’s index to study spatial correlation, the local agglomeration distribution characteristics of the EFI are the main characteristics of “low-low” and “high-high” agglomeration. The results show that the overall EFI spatial distribution of Jiangsu’s counties exhibited agglomeration features and changing characteristics. It can be seen from the results that the agglomeration effect between high and high values and between low and low values is obvious. There were assimilation impacts of counties with strong agglomeration on adjacent units. The reason for the formation of different EFI agglomeration types is contributed by the agglomeration of similar development levels in economic development, industrial structure and green technology.
